# Antibody-Mediated Rejection in Kidney Transplantation: A Review

**DOI:** 10.1155/2012/193724

**Published:** 2012-04-10

**Authors:** Chethan Puttarajappa, Ron Shapiro, Henkie P. Tan

**Affiliations:** ^1^Renal-Electrolyte Division, Department of Medicine, University of Pittsburgh Medical Center, Pittsburgh, PA 15213-2582, USA; ^2^Division of Transplantation, Department of Surgery, University of Pittsburgh Medical Center, Pittsburgh, PA 15213-2582, USA

## Abstract

Antibody mediated rejection (AMR) poses a significant and continued challenge for long term graft survival in kidney transplantation. However, in the recent years, there has emerged an increased understanding of the varied manifestations of the antibody mediated processes in kidney transplantation. In this article, we briefly discuss the various histopathological and clinical manifestations of AMRs, along with describing the techniques and methods which have made it easier to define and diagnose these rejections. We also review the emerging issues of C4d negative AMR, its significance in long term allograft survival and provide a brief summary of the current management strategies for managing AMRs in kidney transplantation.

## 1. Introduction

Antibody-mediated rejection is an important cause of acute and chronic allograft dysfunction and graft loss. Although hyperacute (i.e., preformed antibody-mediated) rejection has been recognized since the 1960s, the role of antibodies in other forms of rejection was not clear until new diagnostic methods became available. Our knowledge about the role of antibodies in allograft rejection, particularly in some forms of chronic allograft rejection, has been evolving rapidly over the last decade.

## 2. Types of Antibody-Mediated Rejection (AMR)

Antibodies directed against donor antigen can cause different types of rejection that can vary in acuity and severity.

**Hyperacute AMR**

It occurs due to preformed donor specific antibodies (DSA) present in high titers and presents as graft failure that can occur within minutes (but sometimes may be delayed for a few days) after transplantation [1]. The occurrence of this type of rejection is extremely rare because of the universal adoption of pretransplantation cross-matching. The histopathology is characterized by features of severe endothelial and arterial injury manifested as arteritis (often transmural), interstitial edema, and severe cortical necrosis, with almost all cases requiring allograft nephrectomy. Most of the initial cases were reported in patients with a history of previous transplantation or in multiparous women, suggesting the role of sensitization and preformed antibodies. Strong proof for this was provided by Patel and Terasaki in 1969 when they showed that 24 of the 30 patients with a pretransplant positive crossmatch had immediate graft failure compared with only 8 graft failures in 195 patients without a positive crossmatch [1].

**Acute AMR**

It is characterized by graft dysfunction manifesting over days and is a result of DSAs, that may either be preformed or develop denovo after transplantation [2]. Acute AMR occurs in about 5–7% of all kidney transplants and is responsible for 20–48% of acute rejection episodes among presensitized positive crossmatch patients [3, 4]. Allograft dysfunction with resultant creatinine elevation may not be present in all cases of AMR. Histopathology in these patients is again related to endothelial injury mediated by antibodies but is less severe than that seen in hyperacute rejections. Biopsy often shows endothelial cell swelling, neutrophilic infiltration of glomeruli and peritubular capillaries, fibrin thrombi, interstitial edema, and hemorrhage [5]. However, in a minority of these rejections, acute tubular necrosis may be the only feature observed [3]. The identification of these AMRs has become easier with the development of C4d-staining in biopsies and improved methods of antibody detection. Prior to the routine use of C4d-staining, diagnosis was often limited by lack of staining for antibody components and was often restricted to steroid-resistant cases with or without obvious histopathologic findings as described above.

**Chronic AMR**

It is now well recognized that antibodies can mediate chronic allograft injury which is characteristically seen as transplant glomerulopathy (TG) on kidney biopsies [6]. TG (also known as or chronic allograft glomerulopathy) is characterized by glomerular mesangial expansion and capillary basement membrane (BM) duplication, seen as basement membrane double contouring or splitting. Similarly, the peritubular capillary (PTC) basement membrane also shows changes, but these are seen mostly on electron microscopy sections as basement membrane multilayering. Clinically, the manifestations range from patients being asymptomatic in the early stages to having nephrotic range proteinuria, hypertension, and allograft dysfunction in the advanced stages. Progression can sometimes be fairly rapid, especially with ongoing acute AMR, resulting in graft failure within months [7]. The prevalence of TG in protocol biopsies has varied between 5% at 1 yr to 20% at 5 years [8].

## 3. Pathogenesis

Antibodies are most commonly directed against human leukocyte antigen (HLA)/major-histocompatibility-complex (MHC) Class I and II antigens [9]. HLA class I antigens are expressed on all nucleated cells, whereas HLA class II antigens are restricted to antigen-presenting cells (B lymphocytes, dendritic cells) and endothelial cells. However, the antibodies can also be directed against other donor specific antigens such as MHC-class I-related chain A (MICA) antigens, MHC-class I-related chain B (MICB) antigens, platelet-specific antigens, molecules of the renin-angiotensin pathway, and polymorphisms involving chemokines and their receptors [10–15]. MICA antigens are expressed on endothelial cells, dendritic cells, fibroblasts, epithelial cells, and many tumors, but not on peripheral-blood lymphocytes. Risk factors for sensitization against HLA I and II antigens are pregnancy, blood transfusions, and previous transplantation. However, blood transfusions were found not to be a risk factor for MICA sensitization [10].

## 4. Mechanisms of Antibody Mediated Injury

The major mechanism involved is activation of classical complement pathway by the antigen-antibody complex, leading to formation of the membrane attack complex resulting in cellular injury. The target antigens in AMR are most often situated on the endothelium resulting in the histological findings of acute (glomerulitis, peritubular capillaritis) and chronic (transplant glomerulopathy) vascular injury. Endothelial damage also results in platelet activation and microthrombi formation. The byproducts of complement activation (e.g., C3a and C5a) act as chemokines resulting in inflammatory cell infiltration and amplification of the inflammatory process. Long standing inflammation results in cell proliferation, basement membrane duplication, and mesangial interposition which can be easily seen on light and electron microscopy as glomerular BM splitting and PTC BM multilayering, respectively. The ability of different IgG subclasses to fix complemsent also varies. IgG1 and IgG3 have strong complement fixing properties compared to the IgG2 and IgG4 subclasses, which fix complement weakly. The significance of this was studied in a series of 74 patients with pretransplant anti-HLA antibodies. Only 4 patients in this series had exclusively weak or no complement fixing HLA antibodies (IgG2 or IgG4) [16]. Of the remaining, 21 and 46 patients had isolated strong complement fixing HLA (IgG1 or IgG3) antibodies or a mix of weak and strong complement fixing HLA antibodies respectively, but had no difference in AMR or graft failure at 5 years. Of the 4 patients with isolated IgG2/IgG4, none had any AMR. Antibodies can also mediate injury via complement independent mechanisms such as antibody-cell-dependent cytotoxicity (ADCC). This is mediated through cells of the innate immunity (natural killer cells, macrophages) which get activated by binding to the Fc receptor portion of the antibody [17]. Antigen antibody interaction on endothelial cells is also known to increase Von Willibrand Factor (vWF) along with externalization of P-selectin molecules resulting in increased platelet activation and leukocyte trafficking, respectively [18, 19].

## 5. Diagnosis of AMR

Based on the increasing evidence for the role of antibodies in allograft dysfunction and the strong correlation with C4d-staining and DSA, the Banff committee updated its renal allograft biopsy classification to involve a separate antibody-mediated rejection diagnosis [20]. According to the classification, AMR was defined as a triad involving the presence of DSA, positive C4d-staining on the biopsy, and histopathological evidence of antibody-mediated injury (glomerulitis, peritubular capillaritis, and arteritis).

Based on the histopathology, AMR can be classified into three subtypes as below.

Class I: Presence of acute tubular necrosis (ATN) only, with minimal inflammation.
Class II: glomerulitis, peritubular capillaritis, and microthrombosis.
Class III: Arteritis.

Chronic AMR according to the Banff criteria involves demonstration of C4d, DSA, and at least one feature of morphologic evidence of chronic tissue injury, such as glomerular double contours, peritubular capillary basement membrane multilayering, interstitial fibrosis/tubular atrophy, and/or intimal thickening of arteries [20]. However, it is not uncommon to have situations where DSAs may be absent even in the presence of histological AMR and positive C4d-staining.

## 6. C4d Stain

C4d is a complement split product that is formed during breakdown of C4b into C4d and C4c. C4d has a thioester moiety that enables strong covalent bonding with the endothelial cells and basement membrane. It is constitutively expressed in all normal kidneys in the mesangium and the vascular pole owing to the constant complement turnover. This can extend into glomerular capillaries in cases of immune-mediated glomerulopathies, but peritubular C4d deposition is noted mostly in the transplanted kidney, with rare reports of C4d presence in PTC of native kidneys [21, 22].

There are two methods for C4d detection in biopsy specimens [23]. It can be detected using either immunofluorescence (IF) on frozen tissue with a monovalent antibody against C4d or using Immunohistochemistry (IHC) on paraffin-embedded tissue with a polyvalent antibody. Diffuse C4d implies >50% of PTC staining for C4d, while focal and minimal staining implies 10–50% and <10% staining, respectively. IHC is less sensitive than IF for C4d detection. Hence, focal staining on IHC may be equivalent to diffuse staining with IF.

The association of C4d with AMR was initially described by Feucht et al. in 1991 when they showed a significant association of C4d with preformed anti HLA antibodies in patients with acute rejection [24]. This was subsequently confirmed in a study of 16 biopsies with DSA and histopathological evidence of AMR (neutrophilic capillaritis). All 16 biopsies with AMR showed diffuse C4d-staining with trace or no staining in the biopsies with acute cellular rejection (*n* = 14) and 5 of the 6 biopsies with cyclosporine toxicity [25]. Crespo et al. evaluated DSA and C4d in steroid-resistant rejections and found positive DSA in 37% of the cases. Among these, 95% had positive PTC C4d-staining [26].

The data for C4d-staining in chronic AMR is more variable, with a few studies showing strong correlation while others showing poor correlation. Mauiyyedi et al. reported presence of C4d in 23 of 38 biopsies with features of chronic AMR (GBM duplication and arterial intimal fibrosis) but no C4d in the control group without features of chronic AMR [27]. Similar strong associations of C4d with TG and peritubular basement membrane multilayering have been described in other studies as well [28, 29]. However, some studies found no correlation between presence of TG and diffuse C4d, with many TG patients showing no C4d positivity [30, 31].

Protocol biopsy studies have also demonstrated another important characteristic of C4d which is variability of staining over time, suggesting a constant flux between states of positive to negative C4d [32]. In a significant number of these biopsies, there was presence of microvascular inflammation (PTC and glomerular capillaritis) in spite of negative C4d-staining [32]. Another recent development has been the identification that certain endothelial transcripts appear to be expressed more often in patients with histological features of AMR even in the absence of C4d-staining. Sis et al. studied a number of endothelial-associated transcripts (ENDAT) in kidney transplants and found that the ENDAT expression was higher in all types of rejection but more so in AMR. Furthermore, about 40% of patients with ENDAT and chronic AMR features demonstrated no C4d-staining. There was also a strong correlation between elevated ENDAT and presence of anti-HLA antibodies, particularly HLA Class II antibodies [33]. The biology of this ENDAT expression appears to be related to the process of endothelial cell activation, repair, and angiogenesis which are well known mechanisms of AMR [33]. This, along with the low sensitivity of C4d for chronic AMR, has given rise to the concept of “C4d-negative AMR” which appears to be as common, if not more than the C4d-positive AMR and has similar poor prognosis in terms of graft survival [33].

## 7. Anti HLA Antibody Detection

The techniques to identify anti HLA antibodies have improved significantly with the development of single-bead antigen testing methods which have very high sensitivity for antibody detection. The complement-dependent cytotoxicity (CDC) still remains the gold standard test for the detection of preformed antibodies prior to transplantation. The addition of antihuman globulin enhances the sensitivity of the assay by cross-linking the antibodies (AHG-CDC). Flow cytometry crossmatch (FXM) assay is more sensitive than the CDC assay and detects antibodies via fluorochrome-tagged antihuman Immunoglobulin antibody. The newer solid phase assays use purified single HLA antigens to detect anti-HLA antibodies by ELISA or flow cytometry techniques. These tests have increased sensitivity to detect presence of anti-HLA antibodies even with a negative FXM [34–36]. The strength of antibody detected in Luminex assays is indicated by Mean Fluorescent Intensity (MFI). However, there is poor standardization of these tests and the MFIs have been found to vary between different centers performing the same test [37]. A further improvement in antibody detection technique was reported by Yabu et al. They tested antibodies for their capacity to fix C1q complement and compared them to regular IgG antibody detection and found a higher specificity for the C1q technique in detecting antibodies associated with TG and poor graft outcomes [38]. This technique may improve our ability to prognosticate the significance of DSAs but will need to be further standardized and validated in a larger patient population.

## 8. Treatment

Although the current diagnosis of AMR requires the concomitant presence of DSA, C4d, and histopathological evidence of AMR, treatment may be initiated in circumstances where the above criteria may not be fulfilled in entirety. This depends on the risk factor profile of the patient (sensitized patient, history of pregnancies, and blood transfusions) and presence or absence of organ dysfunction. Treatment is often initiated in situations of diffuse C4d positivity with allograft dysfunction even in the absence of DSA or histological evidence of AMR. The inability to measure DSAs in these cases may be related to the presence of non anti-HLA antibodies, to antigen not present on the single-bead assays, or to the possibility that the DSAs may be completely adsorbed onto the allograft [39]. Similarly, patients with positive DSA and histological evidence of AMR may not demonstrate any C4d activity, and treatment is often initiated in these patients as well, especially in the presence of allograft dysfunction. C4d in such cases of acute AMR may be negative for a number of reasons. Immunohistochemistry (IHC) is known to be less sensitive compared to immunofluorescence (IF) staining [22, 23]. Also, areas with necrosis may stain falsely negative for C4d and hence, care must be taken to ensure that viable areas of the biopsy specimen are stained for C4d [22].

There is a paucity of randomized controlled trials in the treatment of AMR. Many of the studies have used historical controls to compare the effectiveness of therapies. There is also bound to be some publication bias related to selective publication of positive studies.

Even with these inadequacies, there has been good progress made in developing treatments for AMR. The primary goal in AMR treatment involves targeting the reduction/removal of DSAs and elimination of the B-cell/plasma cell population responsible for the production of these antibodies.

A number of treatment modalities have been employed for the treatment of AMR as characterized below.

1. Antibody removal/neutralization: plasmapheresis, immunoadsorption, intravenous immunoglobulin, and splenectomy.
2. Anti B-Cell therapies: Mycophenolate mofetil, Rituximab, IVIG, and splenectomy.
3. Antiplasma cell therapy: Bortezomib.
4. Anti-T-cell therapies: T-cell depleting agents such as Antithymocyte globulin (ATG).
5. Conversion to tacrolimus-based regimens.
6. Terminal-complement pathway inhibitor: Eculizumab.

The presence of a vast array of therapeutic modalities signifies the ineffectiveness of one drug or one particular combination therapy to reverse or treat AMR successfully in all scenarios. All of these treatments have been used in different combinations by different groups without a good control arm, resulting in poor evidence to argue for the superiority of one treatment regimen. These treatment modalities are also used for pretransplantation desensitization protocols to abrogate positive crossmatch in highly sensitized patients.

### 8.1. Intravenous Immunoglobulin (IVIG)

This is the most commonly used agent either alone or often, in combination with plasmapheresis. Although the exact mechanisms involved are not clear, they appear to involve multiple processes such as neutralization of complement fixing antibodies, alteration in the activity of complement, modulation of Fc receptor activation and function, and regulation of T and B lymphocytes [40]. Recent research has elucidated the possible role in this immunomodulation, for a specific subtype of IgG which possesses sialylated glycan residues near the Fc receptor [41]. These sialylated IgGs were shown to bind to lectin receptor SIGN-R1 or DC-SIGN leading to increased expression of inhibitory Fc receptor (FcR), FcgammaRIIb on inflammatory cells, thereby attenuating inflammation [41, 42]. IVIG is routinely used in one of two doses: high (2 gm/kg) or low (100 mg/kg per session). Low-dose IVIG is mostly used in combination with plasmapheresis where it may help replenish depleted IGs. Initial studies used IVIG at high-doses without plasmapheresis and described a fair degree of success in desensitization prior to transplant and also for treating antibody-mediated rejection [43, 44]. IVIG is generally safe and well tolerated in most patients with occasional side effects such as aseptic meningitis, volume overload, and rarely acute kidney injury possibly related to high osmotic load. Sucrose-based IVIG preparation is to be avoided, while glycine-based preparations are relatively safe.

### 8.2. Plasmapheresis (PP)

Plasmapheresis is very effective in reducing the antibody load but needs to be used in conjunction with other therapies that target the antibody producing mechanisms. The most common type of Plasmapheresis performed is plasma exchange, with albumin being the most common replacement fluid used. It is usually performed on alternate days with a 1–1.5 volume exchange with albumin (commonly) or fresh frozen plasma. Most institutions also follow each PP session with low-dose IVIG (100 mg/kg) [45]. DSAs are monitored along with renal function to document the effectiveness of the therapy. Treatment, if successful, is continued until the level of antibodies has dropped to safe levels along with improvement in renal function. One of the early studies using this combination to successfully reverse humoral rejection was from Montgomery et al. [46]. The same group subsequently used this combination therapy successfully to reduce pretransplant DSA titers in sensitized patients to allow successful transplantation [46].

A retrospective study analyzed one-year graft outcomes of 16 patients with AMR treated with PP and IVIG and 43 ACR patients and found similar overall graft survival of 81% and 84%, respectively, indicating the effectiveness of these therapies in improving outcomes of acute AMR [47]. 

Plasmapheresis is generally well tolerated. Side effects are relatively uncommon and are related to the use of vascular access (infections, bleeding), volume removal, type of replacement fluid used (coagulopathy, hypovolemia, allergic reactions and a small risk of blood borne infection transmission), hypocalcemia, and side effects related to use of anticoagulants [48].

### 8.3. Immunoadsorption with Protein A (IA)

IA is currently not used in the United States. 

IA was studied in a randomized controlled trial in Europe where it proved very successful in reversing severe AMR [49]. Both arms underwent a switch to tacrolimus from cyclosporine, along with treatment for ACR (steroids/ATG) as needed. The study was initiated at a time when PP and IVIG were still not universally used for AMR treatment.

The study was stopped early because of significant success rate in the IA group (80% versus 20%). It was, however, a small study (5 patients in each group), with a higher prevalence of diffuse C4d in the control group. Considering the widespread acceptance of IVIG and PP for treatment of AMR and unavailability of IA in the USA, a head-to-head study of IA with PP and IVIG will be necessary to prove its superiority, prior to its acceptance as an alternative to IVIG and PP.

### 8.4. Rituximab

Rituximab is an anti-CD20 monoclonal antibody that induces profound depletion of B-cells and was initially approved for the treatment of B-cell lymphoma. It has since been tested in multiple immune-mediated disorders with varying degrees of success. Rituximab has been used to treat AMR in a number of uncontrolled studies.

Most of the studies reported so far with the use of Rituximab have reported favorable outcomes. Becker et al. treated 27 patients with AMR with a single dose of rituximab. Twenty-two of these patients also received ATG and plasmapheresis [50]. At a mean of 605 days of followup, only 3 grafts were lost to rejection. Faguer et al. also reported 81% graft survival at 20 months in 8 patients with the use of 4 doses of Rituximab along with PP, mycophenolate, tacrolimus and steroids [51]. Kaposztas et al. reported their experience with use of Rituximab in combination with PP. Twenty-six patients were treated with Rituximab along with PP and IVIG [52]. The graft outcomes were compared to historical controls who had been treated with PP ± IVIG alone. The two-year graft survival for patients treated with rituximab plus PP was significantly better at 90% when compared to the 60% survival in the PP cohort. However, the doses of IVIG were higher in the Rituximab group, and the use of IVIG was also statistically associated with a better graft outcome on Kaplan-Meier analysis, raising concerns for a confounding effect. Lefaucheur et al. also compared the use of 2-week doses of Rituximab along with high-dose IVIG and PP with historical controls who had received high-dose IVIG alone and reported a 91.7% graft survival, compared to 50% with high-dose IVIG alone [53]. The mechanism of action of Rituximab in AMR is not clear, given that the plasma cells do not express CD20 on their surface. However, the depletion of CD20-positive subset of B-cells may attenuate the antibody generation process. The standard dosing of Rituximab is 375 mg/m^2^/wk for 2–4 weeks. Rituximab results in prolonged and profound B-cell depletion which may cause reactivation of latent viruses such as hepatitis B, C, cytomegalovirus (CMV), and also mycobacterium tuberculosis. It also carries a boxed warning for progressive multifocal leukoencephalopathy (PML) caused by JC virus. 

Patients can also manifest acute infusion reactions, which usually occur within 30–120 minutes and may be mild or severe, such as bronchospasm, angioedema, acute respiratory distress syndrome, cardiogenic shock, and anaphylaxis. These have often been reported in leukemic patients with high pretherapy leukocyte counts [54].

### 8.5. Change of Maintenance Immunosuppression (IS)

Initiation or augmentation of anti B-cell maintenance therapy is routinely done when AMR is identified. The most commonly used agent for this purpose is mycophenolate mofetil. It is also common practice to change to a calcineurin-based immunosuppression, specifically to tacrolimus, if patients are not on a calcineurin inhibitor (CNI).

### 8.6. Bortezomib

Bortezomib is a novel proteosome inhibitor that is approved for the treatment of multiple myeloma. Proteasomes are involved in breakdown of ubiquitinated proteins and are present both in the nucleus and cytoplasm. Inhibition of proteasomes can lead to decreased nuclear factor-Kappa B activation, cell cycle arrest, endoplasmic reticulum stress, and increased cell apoptosis [55]. This action is pronounced in plasma cells likely because of the high antibody turnover and high endoplasmic reticulum activity. A number of groups have investigated the use of bortezomib in solid organ AMR and have generally reported favorable results. There is, however, no randomized trial thus far and in most cases, bortezomib was used after standard therapies for AMR failed, that are, IVIG and PP [56–63]. Many similar case series and case reports continue to be reported with good outcomes in AMR with bortezomib. However, one study reported patients with subclinical AMR and positive DSAs in whom bortezomib monotherapy did not result in any significant reduction of DSA levels [64]. The authors and the editorial caution against the use of bortezomib as primary therapy for AMR without strong evidence from randomized studies. However, this agent appears to be a promising strategy and its role in AMR is still evolving. Gastrointestinal side effects, neuropathy, and hematological toxicity are the main side effects of bortezomib and need to be carefully monitored. Dosing in most of these reports has been the standard myeloma dosing of 1.3 mg/m^2^/week, with 4 doses given over 2 weeks.

### 8.7. Eculizumab

Eculizumab is a humanized monoclonal antibody directed against complement protein C5. It binds to the C5 protein with high affinity, thereby inhibiting conversion of C5 to C5b and preventing formation of the membrane attack complex (C5–9). Initially approved for use in paroxysmal nocturnal hemoglobinuria (PMH), it was also recently approved for use in atypical hemolytic-uremic syndrome. Prior vaccination against meningococcus and pneumococcus is necessary. One dose of Eculizumab was used with IVIG and rituximab in a patient with severe AMR, who recovered from the AMR but died of a fatal pulmonary hemorrhage a few months later [65]. A prospective study compared the outcomes of using eculizumab to prevent acute AMR and TG after transplantation in a series of HLA-sensitized pretransplant positive-FXM patients (*n* = 26). The incidence of AMR at 3 months was significantly less compared to an historical control group (7.7% versus 41.2%), although the presence of C4d in patients with DSA did not differ between the study and control group, thus providing evidence for the downstream activity of eculizumab in blocking the complement pathway. The use of eculizumab also resulted in a reduced need for PP. The one-year TG prevalence was also low with only one (6.7%) of the 15 patients in the eculizumab group developing TG compared to 15 (35.7%) of the 42 controls. Most patients in the treatment group received weekly eculizumab for 4–8 weeks while one patient needed a year of eculizumab because of persistent FXM positivity [66]. No significant complications were reported during the study period. Although not a randomized study, this series serves as a proof of concept for the use of terminal complement pathway inhibitors in treating AMR. A major limitation for its use at the present time is its extremely high cost. However, a multicenter prospective randomized trial is being planned to study its efficacy and should help answer some of the questions regarding its role in AMR.

*Splenectomy *It has also been used in resistant AMR patients with good success rate [67, 68]. However, because of the long term risk of infections in immunosuppressed individuals and the surgical risks involved, this is not a commonly used therapy for AMR.

Acute cellular rejection frequently coexists with AMR and needs to be treated aggressively with either steroids or T-cell depleting therapies such as Antithymocyte globulin (ATG). The role of these T-cell depleting therapies in AMR has not been clearly studied in patients with pure AMR or AMR with low grades of ACR. The use of these agents may reduce the T-cell stimuli that are driving the B-cell-mediated antiallograft responses, thereby helping to gain better control of the AMR process. Indirect proof can be obtained from one study that reported no significant change in graft outcomes between C4d-positive and negative cases. However, there was aggressive use of antilymphocyte therapy to treat C4d-positive cases which might have improved outcomes in this group of patients [69].

## 9. Course and Prognosis

It has been shown in multiple studies that AMR portends worse outcome in terms of graft survival at one and five years. Some of these studies were reported prior to the utilization of aggressive therapies that are currently in use for AMR and often serve as historical controls for trials of newer therapies for AMR. Lederer et al. reported a 4 year 50% graft survival for C4d+ patients compared to a 8 year 50% graft survival for C4d-patients [70]. Poduval et al. reported a one year graft loss of 65% for grafts with diffuse C4d+ diagnosis compared to 33% for focal and negative C4d grafts [71]. One study, however, noted no difference between C4d+ and C4d− grafts with up to 3 years followup. However, patients with C4d+ were treated more aggressively with antilymphocytic therapy (ATG and OKT3) [69].

The significance of C4d+ allograft biopsies appears to differ based on whether the transplantation was ABO or HLA incompatible [72]. Multiple studies have documented presence of diffuse C4d with no allograft dysfunction and with no histologic evidence of AMR in protocol biopsies of ABO incompatible transplants [73, 74]. The presence of C4d in these patients was also shown to have no adverse outcome and in fact was associated with a trend toward better scores of chronicity and less TG at subsequent followup [73]. In contrast, the presence of diffuse C4d+ in ABO compatible HLA-mismatched kidney transplantation appears to be very commonly associated with neutrophil margination suggesting ongoing antibody-mediated rejection [72].

The significance of focal C4d on biopsies is currently still being evaluated and is not entirely clear. A significant number of these biopsies may not be associated with histologic evidence of AMR, but its presence has still been associated with inferior graft outcomes [75, 76]. Graft outcomes with a diagnosis of TG are poor, with graft survival of 60% at 5 years compared to >90% without TG [8].

## 10. Management of Patients with Pretransplantation HLA Sensitization

Improvements in HLA typing and DSA identification have increased our ability to identify high-risk recipients who may be at risk for antibody-mediated rejection posttransplantation. For patients who are highly sensitized or those with ABO or HLA incompatible living donors, there are at present four options available for successful transplantation. Patients can undergo desensitization protocol followed by a kidney transplant provided they can achieve sufficient reductions in DSA titers and become crossmatch negative. Even with successful transplantation after desensitization, these patients remain at increased risk for AMR. They also have reduced graft survival compared to nonsensitized patients. However, their outcomes are still superior when compared to remaining on dialysis [77]. Patients can also undergo a paired living kidney donation (PKD) involving 2 or more donor recipient pairs. Another potential option less commonly used is List paired donation (LPD) which involves the option of a recipient with an incompatible living donor getting a deceased donor kidney from a waiting list in return for the living donor donating it to the intended recipient on the transplant waiting list [78]. The least favorable option would be to remain on the deceased donor list waiting for a compatible donor. However, for some highly sensitized patients with living donors, transplantation by paired exchange or list-paired donation may not be possible [78]. These patients should preferably undergo desensitization to improve the likelihood of transplantation which, as mentioned earlier, has been shown to offer improved patient survival compared to waiting on the transplant list [77].

## 11. Summary

Antibody-mediated rejection is an important cause of acute and chronic graft failure. Improvements in HLA technology along with the recognition of the role of C4d in AMR have revolutionized the understanding of this important entity. New research is attempting to elucidate the mechanisms and epidemiology of C4d-negative antibody-mediated rejection processes. Further research should help clarify the identification, prognosis, and treatment of these C4d-negative AMRs. Therapies for AMR are still not optimal with high rates of graft loss leading to poor patient outcomes. Newer therapies, such as bortezomib and eculizumab that target novel pathways in the AMR process are promising but will need further randomized studies before becoming widely used. Studies will need to be performed to determine the best use, either alone or in combination, of the myriad number of therapies currently available. Transplantation of sensitized patients remains a difficult problem. However, developments such as paired kidney donation and desensitization protocols are continuously improving the rates of transplantation in this difficult to transplant population.
